# Mortality and admission to intensive care units after febrile neutropenia in patients with cancer

**DOI:** 10.1002/cam4.2955

**Published:** 2020-03-07

**Authors:** Theis Aagaard, Joanne Reekie, Mette Jørgensen, Ashley Roen, Gedske Daugaard, Lena Specht, Henrik Sengeløv, Amanda Mocroft, Jens Lundgren, Marie Helleberg

**Affiliations:** ^1^ Centre of Excellence for Health, Immunity and Infections (CHIP) Rigshospitalet University of Copenhagen Copenhagen Denmark; ^2^ Centre for Clinical Research, Epidemiology, Modelling and Evaluation (CREME) Institute for Global Health University College London London UK; ^3^ Department of Oncology Rigshospitalet University of Copenhagen Copenhagen Denmark; ^4^ Department of Haematology Rigshospitalet University of Copenhagen Copenhagen Denmark

**Keywords:** cancer, febrile neutropenia, infection, mortality, prognosis

## Abstract

Febrile neutropenia (FN) is a critical complication of chemotherapy associated with increased in‐hospital mortality. However, associations with increased mortality and intensive care unit (ICU) admissions during longer follow‐up are not established. Patients treated with standard first‐line chemotherapy for solid cancers at Rigshospitalet, Denmark in 2010‐2016 were included. Incidence rate ratios (IRR) of all‐cause, infectious and cardiovascular mortality, and ICU admissions after FN were analyzed by Poisson regression. Risk factors at the time of FN were analyzed in the subpopulation of patients with FN; all‐cause mortality was further stratified by the time periods 0‐30, 31‐365, and 366+ days after FN. We included 9018 patients with gastric (14.4%) and breast (13.1%) cancer being the most common, 51.2% had locally advanced or disseminated disease and the patients had a median Charlson Comorbidity Index score of 0 (interquartile range, 0‐0). During follow‐up, 845 (9.4%) experienced FN and 4483 (49.7%) died during 18 775 person‐years of follow‐up. After adjustment, FN was associated with increased risk of all‐cause mortality, infectious mortality, and ICU admissions with IRRs of 1.39 (95% CI, 1.24‐1.56), 1.94 (95% CI, 1.43‐2.62), and 2.28 (95% CI, 1.60‐3.24). Among those with FN, having a positive blood culture and low lymphocytes were associated with excess risk of death and ICU admissions (predominantly the first 30 days after FN), while elevated C‐reactive protein and low hemoglobin predicted mortality the first year after FN. The risk of death varied according to the time since FN; adjusted IRR per additional risk factor present for the time periods 0‐30, 31‐365, and 366+ days after FN were 2.00 (95% CI, 1.45‐2.75), 1.36 (95% CI, 1.17‐1.57), and 1.17 (95% CI, 0.98‐1.41). FN was associated with increased mortality and risk of ICU admissions. An objectively identifiable subgroup of patients among those with FN carried this excess risk.

## Graphical abstract

Febrile neutropenia (FN) is a severe complication to chemotherapy. However, associations between FN and longer term clinical outcomes are poorly elucidated. Among 9018 patients with solid cancers followed for 18 775 person‐years after initiating first‐line chemotherapy FN was associated with increased risk of all‐cause and infectious mortality and risk of intensive care unit admissions. A range of risk factors at the time of FN were found that identified a subgroup of patients carrying a grave prognosis.

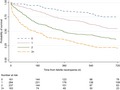

## 1 INTRODUCTION

Febrile neutropenia (FN) is a critical complication of chemotherapy developing in 7.9%‐11.7% of patients.^1^, ^2^ FN is associated with increased morbidity^3^, ^4^, ^5^ and an in‐hospital mortality rate around 10%^4^, ^6^ and is a major dose‐limiting event occurring during chemotherapy in patients with cancer.^1^, ^4^, ^7^ Risk factors for short‐term complications of FN (death, organ failure, or admission to the intensive care unit [ICU]) include age, cancer type, comorbidities, delayed antibiotics, and laboratory or vital sign abnormalities.^5^, ^8^, ^9^, ^10^ Different combinations of these risk factors have been used to develop the Talcott, MASCC, and CISNE risk scores^8^, ^9^, ^10^ that have contributed widely to improve the management of FN. However, there are limited data on longer term outcomes after FN and their associated risk factors. Besides one study that found increased risk of hospitalizations and mortality after FN,^11^ and another looking at the increased long‐term risk of infections in surviving patients who experienced FN during chemotherapy,^12^ there is limited evidence on clinical outcomes after FN on the longer term. The effect of FN on mortality is also uncertain and may depend on several mechanisms that vary over time: First, by infection‐related immediate mortality^6^; second, by subsequent chemotherapy dose delays and dose reductions that result in lower relative dose intensity and thus, increased long‐term mortality^4^; and third, by inflicting organ damage leading to increased noninfectious mortality, both short^13^ and long terms.^14^ Altogether, risk factors in relation to FN that are associated with increased morbidity and mortality are important to identify, both on short and long terms, to improve and strengthen the evidence base for clinical monitoring and prophylactic interventions.

Our main hypothesis was that we would find increased risk of ICU admissions and all‐cause mortality after FN, and that both the infectious and cardiovascular mortality would be increased. In addition to this, we aimed to identify risk factors at the time of FN associated with increased risk of mortality and ICU admissions and investigate whether their effects were evident in both short and long terms.

## 2 METHODS

### 2.1 Study design and patient selection

We performed a retrospective cohort study of treatment‐naïve patients with cancer from the Department of Oncology at Rigshospitalet, University of Copenhagen, who initiated their first cycle of chemotherapy between 1 January 2010 and 30 November 2016. Rigshospitalet is a tertiary center with varying catchment areas for each cancer. For example, gastric cancer was the most common cancer identified in this study due to a large catchment area.

To be eligible for the study, patients had to be treated with standard first‐line chemotherapy. As per previous analyses of the cohort,^15^, ^16^ patients with temporary civil registration numbers, patients registered as initiating two different chemotherapy regimens simultaneously, and patients with stem cell transplantations were excluded from the analysis. We further excluded patients who received oral‐only chemotherapy for whom we did not have data on dose delays and dose reductions as we found these data essential for the analyses.

Patients in our institution are treated according to commonly used international standards for each cancer group and disease stage. Prophylactic antibiotics are generally discouraged and patients presenting with FN are treated according to the European Society of Medical Oncology (ESMO) guidelines.^17^ The empiric antibiotics of choice were cephalosporins in combination with gentamicin until October 2014 and piperacillin/tazobactam in combination with gentamicin afterward. Carbapenems were used in case of allergy and gentamicin was substituted with ciprofloxacin in patients treated with cisplatin. Amifostine is not used in Denmark.

The study was approved by the Danish Data Protection Agency (2012‐58‐0004; RH‐2016‐47; 04433) and the Danish National Board of Health (3‐3013‐1060/1/).

### 2.2 Data sources

We employed data from the data repository at the Centre of Excellence for Personalised Medicine for Infectious Complications in Immune Deficiency (PERSIMUNE). The repository contains data from electronic health records, including nationwide data on biochemistry and microbiology and regional data on medication.^15^ Further, we used data from the Danish Civil Registration System on mortality, emigration, and loss to follow‐up^18^ and data from the National Patient Register^19^ containing dates, diagnoses, and procedure codes for inpatient and outpatient services. Finally, we used data from the Danish Register of Causes of Death^20^ (data for 2016 were not available, and hence, patients who died in 2016 were excluded from analyses of infectious and cardiovascular mortality). These data sources were linked using the unique 10‐digit civil registration number given to all Danish citizens. The data that support the findings of this study are available from the corresponding author upon reasonable request.

### 2.3 Outcomes

The primary outcome was all‐cause mortality. The secondary outcomes were infectious and cardiovascular mortality and ICU admissions. We defined the cause of death to be infectious or cardiovascular if the underlying cause of death or one of the three contributory causes of death listed on the death certificate were infectious or cardiovascular. Therefore, the same patient could be registered with both infectious and cardiovascular mortality. Details can be found in the Supplementary Material.

### 2.4 Primary exposure

The primary exposure was FN during the first chemotherapy course. FN was defined as in previous studies^15^, ^16^ as a blood culture (regardless of whether it was positive or negative) or death within three days of a neutrophil count <0.5 × 10^9^/L or a leucocyte count ≤2.0 × 10^9^/L if neutrophils were not measured. In our setting, blood cultures are collected when there is fever or clinical suspicion of infection. Further, blood cultures are not sampled for routine surveillance in neutropenic patients. Data on temperature measurements were not routinely available before 2014 and hence a blood culture was used as a measure of clinical suspicion of infection. In previous studies we have shown that 85%‐89% of patients identified by our definition have a fever of 38 degrees Celsius or higher. Further, we have found that only 4.5%‐6.5% of patients identified with a strict definition of FN (defined as fever ≥38 degrees Celsius within three days of a neutrophil count <0.5 × 10^9^/L) were missed by our definition.^15^, ^16^ As mortality was our primary outcome, patients identified as experiencing FN through death, with no blood culture, were excluded.

### 2.5 Statistical methods

Associations between FN and mortality or ICU admissions were examined by Poisson regression. Patients were included from the date of chemotherapy initiation and followed until, death, emigration, loss‐to follow‐up, or 31 December 2016, whichever came first. For the secondary outcome of ICU admissions, follow‐up ended at the date of admission to the ICU.

Febrile neutropenia was included as a time‐updated variable. All patients were initially categorized in the no FN group and contributed person‐time to this category until they experienced their first FN event as defined above. If a patient experienced FN, the patient then contributed person‐time to the FN group from the date of FN until the end of follow‐up. The following variables assessed at the time of chemotherapy initiation were adjusted for in the model: Sex, age, and comorbidity as measured by the Charlson Comorbidity Index score^21^, ^22^ (calculated without the contributions from cancer), cancer type, disease stage, calendar year, history of radiation, body surface area, and anemia. Treatment with granulocyte colony‐stimulating factors (G‐CSF), chemotherapy dose delay and dose reduction, and number of chemotherapy cycles received during the first‐line treatment were included as time‐updated risk factors (for details on variables, see the Supplementary Material).

In analyses of risk factors for death and ICU admission after FN, we included only patients who experienced FN and followed them from the date of their first FN event. The incidence rates (IRs) of mortality (all‐cause, infectious, and cardiovascular) and ICU admission were stratified by time periods since FN (0‐30, 31‐365, and 366+ days). We used Poisson regression analyses to investigate risk factors at chemotherapy initiation and the following risk factors at the time of the FN event: blood culture result (positive/negative),^23^ C‐reactive protein (CRP) and hemoglobin levels, and platelet and lymphocyte counts. A significant interaction was found between the time periods since FN and several of the risk factors. For the main outcome, all‐cause mortality, the model was therefore stratified into these time periods. Lastly, patients were grouped according to the number of significant risk factors (defined as a *P* < .1 in univariable analyses) present at the time of FN and the impact on all‐cause mortality per additional risk factor present was assessed by Poisson regression. We used the median value for each risk factor at the time of FN as the cutoff for presence of that risk factor. Presence of the risk factors was thus defined as a positive blood culture, CRP ≥79 mg/L, hemoglobin ≤10.6 g/dL, and lymphocytes ≤600/µL.

Continuous variables were assessed in quartiles and the quartiles were collapsed based on visual inspection of Kaplan‐Meier plots of all‐cause mortality after FN. Missingness was included as a separate category for each variable. Due to low frequencies of FN (n < 30), patients with cancer of the central nervous system, esophagus, mesothelium, colon, rectum, cervix, endometrium, and bladder were collapsed in the other cancer group for statistical analyses of the subpopulation of patients who experienced FN.

Statistical analyses were performed with STATA (StataCorp. 2017. *Stata Statistical Software: Release 15*; StataCorp LLC) and SAS (Version 9.4, SAS Institute, Inc).

### 2.6 Sensitivity analyses

To test the robustness of our results, we did a sensitivity analyses where we assessed FN events occurring in any chemotherapy course during the entire study period and not only FN events occurring during the first chemotherapy course.

## 3 RESULTS

**FIGURE 1:**
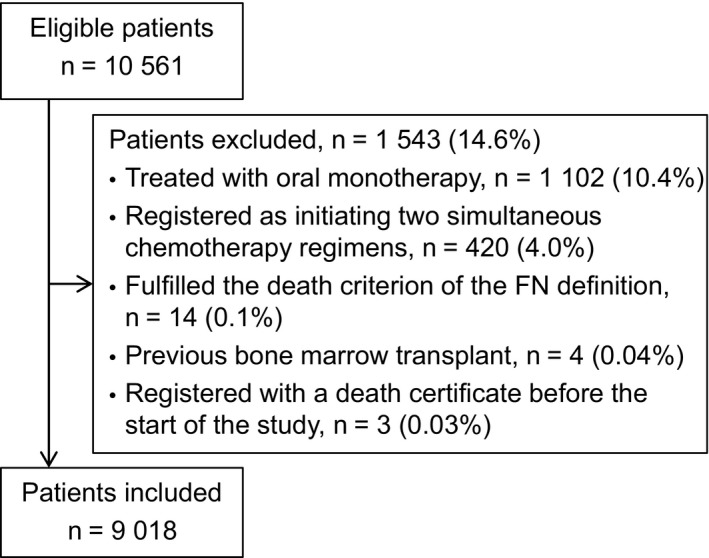
Flow diagram for inclusion of patients with cancer initiating standard first‐line chemotherapy in 2010‐2016

During the 7‐year study period we identified 10 561 patients with valid civil registration numbers who initiated standard first‐line chemotherapy of whom 9018 were included in our study. Reasons for exclusion are presented in Figure 1. The most frequent cancers were gastric (n = 1298, 14.4%), breast (n = 1182, 13.1%), and nonsmall‐cell lung cancer (n = 1076, 11.9%). The median age was 63.8 (interquartile range [IQR], 54.3‐70.6), 4517 (50.1%) were male, 4614 (51.2%) had locally advanced or disseminated disease and the patients had a median Charlson Comorbidity Index score of 0 (IQR, 0‐0). Baseline characteristics for all patients and for the subgroup of patients who experienced FN are presented in Table 1.

**TABLE 1:** Characteristics of all patients and the patients who experienced febrile neutropenia during the first chemotherapy course in patients with solid cancers, 2010‐2016

	All patients	Patients with FN
Patients, n (%)	9018 (100)	845 (9.4)
Male sex, n (%)	4517 (50.1)	348 (41.2)
Cancer type, n (%)		
Ovarian	577 (6.4)	131 (15.5)
Breast	1182 (13.1)	124 (14.7)
Nonsmall‐cell lung	1076 (11.9)	124 (14.7)
Gastric	1298 (14.4)	96 (11.4)
Small‐cell lung	324 (3.6)	84 (9.9)
Testicular	316 (3.5)	55 (6.5)
Prostate	285 (3.2)	45 (5.3)
Neuroendocrine	265 (2.9)	43 (5.1)
Head and neck	624 (6.9)	41 (4.9)
Esophageal	425 (4.7)	27 (3.2)
Mesothelioma	489 (5.4)	17 (2.0)
Bladder	276 (3.1)	14 (1.7)
Colon/rectal	1019 (11.3)	12 (1.4)
Cervical/endometrial	488 (5.4)	8 (1.0)
Central nervous system	62 (0.7)	1 (0.1)
Other	312 (3.5)	23 (2.7)
Disease stage, n (%)		
Adjuvant	1807 (20.0)	138 (16.3)
Neoadjuvant or concomitant	2597 (28.8)	155 (18.3)
Locally advanced or disseminated	4614 (51.2)	552 (65.3)
History of radiation, n (%)	1187 (13.2)	112 (13.3)
Body surface area >2 m^2^, n (%)	2127 (23.6)	165 (19.5)
Anemia at baseline, n (%)^a^	3319 (36.8)	355 (42.0)
Age (years), median (IQR)	63.8 (54.3‐70.6)	64.4 (54.3‐70.7)
Charlson Comorbidity Index, median (IQR)^b^	0 (0‐0)	0 (0‐1)
Calendar year, median (IQR)	2013 (2011‐2015)	2013 (2011‐2015)
Cycle n (per patient), median (IQR)^c^	4 (2‐6)	4 (3‐6)

Abbreviations: FN, febrile neutropenia; IQR, interquartile range.

^a^ The reference range differs based on sex and age; see Supplementary Material for details.

^b^ Calculated without the contributions from cancer.

^c^ During the first chemotherapy course.

Of the 9018 patients included in the study, 845 (9.4%) experienced FN during their first line treatment (see Table S1 for a list of the most common regimens), with 453 (53.6%) FN events occurring in the first cycle. Prophylactic G‐CSF was used in 1198/9018 (13.3%) patients with the majority being patients with breast cancer (825/1198, 68.9%). Death occurred in 4483 patients (49.7%) during 18 775 PYFU (IR per 100 PYFU = 23.9, 95% CI, 23.2‐24.6). The crude incidence rate ratios (IRR), comparing those with FN to those without, were higher for all‐cause (IRR 1.44, 95% CI, 1.28‐1.62, *P* < .0001), infectious mortality (IRR 1.95, 95% CI, 1.49‐2.55, *P* < .0001), and cardiovascular mortality (IRR 1.52, 95% CI, 1.01‐1.52, *P* = .047) (Table 2). After adjustment, FN remained associated with an increased risk of all‐cause mortality (adjusted IRR 1.39, 95% CI, 1.24‐1.56, *P* < .0001) and infectious mortality (adjusted IRR 1.94, 95% CI, 1.43‐2.62, *P* < .0001), but not cardiovascular mortality (IRR 1.39, 95% CI, 0.88‐2.20, *P* = .16). ICU admissions occurred in 331 patients (3.7%) during 18 506 PYFU (IR per 100 PYFU = 1.8, 95% CI, 1.6‐2.0). Patients who experienced FN had a twofold higher risk of ICU admissions (adjusted IRR 2.28, 95% CI, 1.60‐3.24, *P* < .0001).

**TABLE 2:** Incidence rates and incidence rate ratios for all‐cause mortality, infectious mortality, cardiovascular mortality, and intensive care unit admissions for patients with and without febrile neutropenia

	Incidence per 100 person‐years (95% CI)	IRR (95% CI)	Adjusted IRR (95% CI)^a^
Mortality			
All‐cause			
FN	33.3 (30.3‐36.3)	1.44 (1.28‐1.62)	1.39 (1.24‐1.56)
No FN	23.1 (22.4‐23.8)	1	1
Infectious^b^			
FN	5.4 (4.1‐6.7)	1.95 (1.49‐2.55)	1.94 (1.43‐2.62)
No FN	2.8 (2.5‐3.0)	1	1
Cardiovascular^b^			
FN	2.0 (1.3‐2.8)	1.52 (1.01‐2.31)	1.39 (0.88‐2.20)
No FN	1.3 (1.2‐1.5)	1	1
ICU admissions			
FN	3.2 (2.3‐4.2)	1.94 (1.4‐2.68)	2.28 (1.60‐3.24)
No FN	1.7 (1.5‐1.9)	1	1

Abbreviations: CI, confidence interval; FN, febrile neutropenia; G‐CSF, granulocyte colony‐stimulating factors; ICU, intensive care unit; IRR, incidence rate ratio; PYFU, person‐years of follow‐up.

^a^ Adjusted for risk factors assessed at chemotherapy initiation: sex, age, comorbidity, cancer type, disease stage, calendar year, history of radiation, body surface area, and anemia at baseline, and the time‐updated risk factors: prophylactic G‐CSF, any chemotherapy dose delay ≥15%, any chemotherapy dose reduction ≥15%, and the number of cycles in the first chemotherapy course.

^b^ N = 8099. We excluded 919 patients with unknown cause of death. Thirty patients were identified with both infections and cardiovascular diseases contributing to cause of death and were included in analyses of both infectious and cardiovascular mortality.

### 3.1 Associations between experiencing FN and risk of death and ICU admissions

Among the 845 patients with FN, death occurred in 472 (55.9%) patients during 1417 PYFU after FN (IR per 100 PYFU, 33.3, 95% CI, 30.3‐36.3). The risk of death was highest in the first 30 days following the FN event (IR per 100 PYFU, 113.7, 95% CI, 87.8‐139.5) with lower IR in the periods 31‐365 days (IR per 100 PYFU, 46.4, 95% CI, 40.6‐52.1) and 366+ days (IR per 100 PYFU, 18.3, 95% CI, 15.3‐21.2) after FN. Compared with the first 30 days after FN, the adjusted relative risk of death was lower in the periods 31‐365 days (adjusted IRR 0.54, 95% CI, 0.42‐0.71, *P* < .0001) and 366+ days (adjusted IRR 0.41, 95% CI, 0.30‐0.56, *P* < .0001) after FN.

High CRP and low hemoglobin levels and low lymphocyte counts at the time of FN were associated with increased risk of all‐cause mortality (Table 3). A significant interaction was observed between the time since FN and positive blood cultures (*P* = .0002), CRP (*P* = .01), and lymphocyte counts (*P* = .046). Consequently, the analyses were stratified by the time periods since FN.

**TABLE 3:** Multivariable analyses of risk factors at the time of febrile neutropenia for all‐cause mortality and ICU admissions after febrile neutropenia

	Number of events	Incidence per 100 PYFU (95% CI)	IRR (95% CI)	Adjusted IRR (95% CI)^a^
All‐cause mortality				
Blood cultures				
Negative	414	31.3 (28.3‐34.3)	1	1
Positive	58	61.2 (45.5‐77.0)	2.0 (1.4‐2.8)	1.0 (0.7‐1.5)
C‐reactive protein				
<37 mg/L	64	13.3 (10.0‐16.5)	1	1
37‐78 mg/L	101	25.5 (20.6‐30.5)	1.9 (1.4‐2.7)	1.3 (0.9‐1.7)
79‐146 mg/L	123	40.6 (33.4‐47.8)	3.1 (2.2‐4.3)	1.4 (1.1‐1.9)
>146 mg/L	169	97.4 (82.7‐112.1)	7.4 (5.3‐10.3)	2.1 (1.5‐2.8)
Missing	15	24.0 (13.4‐39.5)	1.8 (1.0‐3.4)	1.2 (0.7‐2.2)
Hemoglobin				
<9.7 g/dL	160	63.7 (53.8‐73.5)	2.9 (2.3‐3.8)	1.3 (1.0‐1.7)
9.7‐10.6 g/dL	129	37.7 (31.2‐44.2)	1.7 (1.3‐2.3)	1.4 (1.1‐1.7)
>10.6 g/dL	175	21.7 (18.5‐24.9)	1	1
Missing	8	46.1 (19.9‐90.8)	2.1 (1.0‐4.6)	1.0 (0.5‐2.0)
Lymphocytes				
<400/µL	132	49.0 (40.6‐57.3)	2.1 (1.6‐2.8)	1.4 (1.1‐1.8)
400‐600/µL	95	41.1 (32.9‐49.4)	1.8 (1.3‐2.4)	1.3 (1.0‐1.7)
>600/µL	169	23.4 (19.9‐26.9)	1	1
Missing	76	39.0 (30.2‐47.7)	1.7 (1.2‐2.3)	1.2 (0.9‐1.6)
ICU admissions				
Blood cultures				
Negative	35	2.7 (1.8‐3.6)	1	1
Positive	11	12.3 (6.1‐22.0)	4.6 (2.1‐9.7)	3.0 (1.3‐7.1)
C‐reactive protein				
<37 mg/L	7	1.5 (0.6‐3.0)	1	1
37‐78 mg/L	10	2.6 (1.2‐4.7)	1.7 (0.7‐4.7)	1.3 (0.5‐3.8)
79‐146 mg/L	9	3.1 (1.4‐5.9)	2.1 (0.8‐5.7)	1.3 (0.5‐3.7)
>146 mg/L	19	11.0 (6.6‐17.2)	7.5 (3.1‐18.3)	2.1 (0.8‐5.5)
Missing	1	1.6 (0.0‐8.9)	1.1 (0.1‐8.7)	0.9 (0.2‐3.9)
Hemoglobin				
<9.7 g/dL	14	5.6 (3.1‐9.4)	2.5 (1.2‐5.1)	1.2 (0.5‐3.0)
9.7‐10.6 g/dL	13	3.9 (2.1‐6.6)	1.7 (0.8‐3.6)	1.2 (0.5‐3.2)
>10.6 g/dL	18	2.3 (1.4‐3.6)	1	1
Missing	1	5.8 (0.2‐7.2)	2.5 (0.4‐17.3)	1.2 (0.2‐5.9)
Lymphocytes				
<400/µL	21	8.0 (4.6‐11.5)	4.8 (2.3‐10.0)	2.9 (1.2‐6.9)
400‐600/µL	5	2.2 (0.7‐5.1)	1.3 (0.4‐3.7)	0.8 (0.2‐2.6)
>600/µL	12	1.7 (0.9‐2.9)	1	1
Missing	8	4.3 (1.8‐8.4)	2.5 (1.0‐6.3)	1.3 (0.4‐4.0)

Abbreviations: CI, confidence interval; ICU, intensive care unit; IRR, incidence rate ratio; PYFU, person‐years of follow‐up.

^a^ Adjusted for the other risk factors in the table and risk factors assessed at chemotherapy initiation: sex, age, comorbidity, cancer type, disease stage, calendar year, history of radiation, and body surface area, and further adjusted for cycle number of the FN event, and time periods after FN.

During the first 30 days after the FN event, both patients with a positive blood culture and patients with low lymphocyte counts had an almost threefold increased risk of all‐cause mortality. During the whole first year after FN, CRP levels exhibited a dose‐response pattern association with all‐cause mortality, and low hemoglobin was also associated with a poor prognosis (Table 4). More than a year after FN, none of the risk factors were significantly associated with risk of all‐cause mortality. Platelet counts were not prognostic (results not shown).

**TABLE 4:** Risk factors at the time of febrile neutropenia for all‐cause mortality stratified by time periods after febrile neutropenia

	0‐30 d, n = 845	31‐365 d, n = 771^a^	366+ d, n = 445^b^
	Adjusted IRR (95% CI)^c^		
Blood cultures (ref: negative)	2.9 (1.5‐5.3)	0.6 (0.3‐1.1)	0.8 (0.5‐1.4)
C‐reactive protein (ref: <37 mg/L)			
37‐78 mg/L	3.2 (0.9‐11.6)	1.4 (0.9‐2.2)	1.0 (0.6‐1.7)
79‐146 mg/L	1.7 (0.4‐6.9)	2.0 (1.3‐3.2)	0.9 (0.6‐1.6)
>146 mg/L	4.8 (1.3‐17.2)	2.4 (1.5‐3.9)	1.3 (0.7‐2.2)
Missing	1.7 (0.2‐17.8)	1.7 (0.8‐3.5)	1.0 (0.4‐2.7)
Hemoglobin (ref: >10.6 g/dL)^d^			
<9.7 g/dL	2.1 (1.2‐3.9)	1.5 (1.1‐2.1)	1.0 (0.6‐1.7)
9.7‐10.6 g/dL	1.4 (0.7‐2.6)	1.6 (1.2‐2.3)	1.2 (0.8‐1.8)
Lymphocytes (ref: >600/µL)			
<400/µL	2.6 (1.2‐5.7)	1.2 (0.8‐1.7)	1.5 (0.9‐2.4)
400‐600/µL	1.7 (0.7‐3.9)	1.2 (0.8‐1.8)	1.6 (1.0‐2.7)
Missing	1.2 (0.4‐3.2)	1.3 (0.9‐2.0)	0.9 (0.5‐1.8)

Abbreviations: CI, confidence interval; FN, Febrile neutropenia; IRR, incidence rate ratio.

^a^ Only patients alive at day 31 after FN were included.

^b^ Only patients alive at day 366 after FN were included.

^c^ Adjusted for the other risk factors in the table and risk factors assessed at chemotherapy initiation: sex, age, comorbidity, cancer type, disease stage, calendar year, history of radiation, and body surface area, and the time‐updated risk factor: cycle number of the FN event.

^d^ Eleven patients with missing values for hemoglobin were included in the reference category after comparing the coefficients from the multivariable model.

**FIGURE 2:**
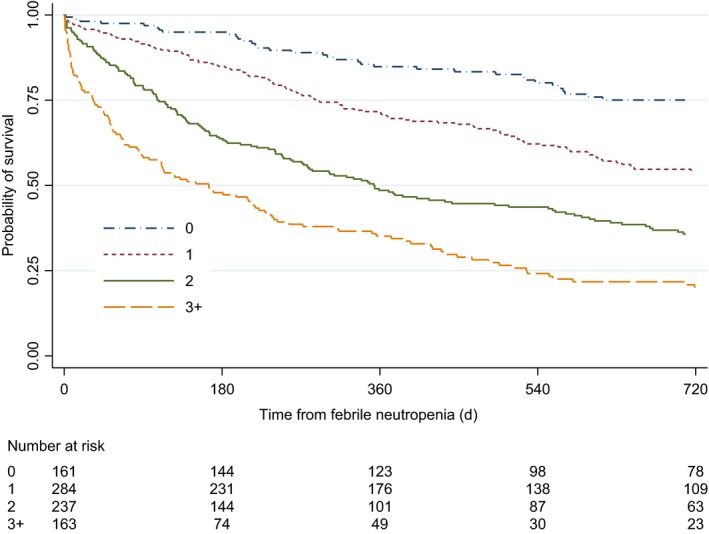
Kaplan‐Meier plot of all‐cause mortality after febrile neutropenia according to the number of risk factors present at the time of febrile neutropenia. Number of risk factors was calculated based on how many of the following the patient had present at the time of febrile neutropenia: positive blood culture, CRP ≥79 mg/L, hemoglobin ≤10.6 g/dL, and lymphocytes ≤600/µL. CRP, C‐reactive protein

All‐cause mortality rates increased according to the number of statistically significant risk factors present (ie, positive blood cultures, high CRP and low hemoglobin levels, and low lymphocyte counts) at the time of FN (Figure 2). Few patients had all four risk factors present, thus we collapsed patients with three and four risk factors as one group. The crude IRR for all‐cause mortality per additional risk factor was 1.92 (95% CI, 1.71‐2.17, *P* < .0001) and the adjusted IRR was 1.35 (95% CI, 1.22‐1.51, *P* < .0001). When stratified by time periods after FN, the adjusted IRR per risk factor present was highest in the period 0‐30 days after FN (adjusted IRR 2.00, 95% CI, 1.45‐2.75), and lower in the periods 31‐363 days (adjusted IRR 1.36, 95% CI, 1.17‐1.57) and 366+ days (adjusted IRR 1.17, 95% CI, 0.98‐1.41) after FN (Table 5 and Figure 2). Among the 747 patients with FN, during the period where cause of death could be determined, 374 (50.1%) patients died, of whom 66 (8.8%) died from infectious mortality, 23 (3.1%) from cardiovascular diseases, and 3 (0.4%) had both infectious and cardiovascular diseases listed as contributory causes of death. Similar trends as described above were observed for infectious mortality, with the highest mortality in the first 30 days after FN and significant associations with high CRP levels and low lymphocytes counts. However, we did not have the statistical power to stratify by time periods after FN. For cardiovascular mortality, estimates were mostly similar to those for infectious mortality but with wider confidence intervals (results not shown).

**TABLE 5:** All‐cause mortality by the number of risk factors present at the time of febrile neutropenia; overall and stratified by time periods after febrile neutropenia

	Overall	Time periods after FN		
		0‐30 d^a^	31‐365 d	366+ d
N	845	845	774	445
N by number of risk factors, 0/1/2/3+	161/284/237/163	3/12/22/37	158/272/218/126	122/174/101/48
Deaths by number of risk factors, 0/1/2/3+	46/140/158/128	3/12/22/37	20/67/96/66	23/61/40/25
Incidence per 100 PYFU (95% CI)				
0	11.7 (8.3‐15.1)	23.0 (4.7‐67.1)	15.5 (9.5‐24.0)	9.1 (5.4‐12.9)
1	25.8 (21.6‐30.1)	53.0 (27.4‐92.5)	32.7 (24.8‐40.5)	19.4 (14.5‐24.3)
2	46.6 (39.3‐53.9)	120.5 (70.1‐170.8)	70.9 (56.7‐85.1)	21.6 (14.9‐28.3)
3+	90.0 (74.4‐105.5)	332.1 (225.1‐439.2)	97.6 (74.0‐121.1)	39.4 (23.9‐54.8)
IRR per additional risk factor (95% CI)	1.92 (1.71‐2.17)	2.51 (1.89‐3.32)	1.82 (1.60‐2.07)	1.52 (1.27‐1.82)
Adjusted IRR per additional risk factor (95% CI)	1.35 (1.22‐1.51)^b^	2.00 (1.45‐2.75)^c^	1.36 (1.17‐1.57)^c^	1.17 (0.98‐1.41)^c^

Abbreviations: CI, confidence interval; FN, Febrile neutropenia; IRR, incidence rate ratio; PYFU, person‐years of follow‐up.

^a^ Patients with head and neck cancer were grouped with the other group since there were no deaths in this group in the period 0‐30 d after febrile neutropenia.

^b^ Adjusted for the risk factors assessed at chemotherapy initiation: sex, age, comorbidity, cancer type, disease stage, calendar year, history of radiation, and body surface area, and further adjusted for time periods after FN.

^c^ Adjusted for the risk factors assessed at chemotherapy initiation: sex, age, comorbidity, cancer type, disease stage, calendar year, history of radiation, and body surface area.

### 3.2 Sensitivity analyses

When we included FN events occurring during several lines of chemotherapy throughout the study period instead of only during the first‐line treatment, we identified 1443 patients with a first‐time FN event. The adjusted IRR for all‐cause mortality after FN was 1.92 (95% CI, 1.74‐2.12, *P* < .0001).

## 4 DISCUSSION

In this cohort study of consecutive patients with cancer treated with standard first‐line chemotherapy with complete follow‐up, we found increased risk of all‐cause and infectious mortality and ICU admissions in patients who experienced FN during first‐line treatment. Further, we identified positive blood cultures, high CRP and low hemoglobin levels, and low lymphocyte counts at the time of FN as independent markers of increased mortality the first year after FN. Presenting with these risk factors most likely reflects a combination of the severity of the FN event and the prognosis associated with the underlying cancer. Consequently, if these results are validated, the grouping of FN events according to this severity grading can be used in future studies. For example, a randomized controlled trial that investigates the effect of an intervention aimed at preventing FN can assess the severity of the FN events in the randomized arms as a secondary outcome.

We found that positive blood cultures were associated with increased 30‐day mortality but not later periods, an association also found in noncancer patients.^24^ During the first 30 days after FN, lymphopenia was also associated with increased risk of all‐cause mortality, in agreement with the findings by Borg et al who further identified the CD4 subpopulation of lymphocytes to be the driver of this association.^25^ Low lymphocyte counts have generally been shown to be associated with a poor prognosis^26^ and we found a moderate correlation between lymphocyte counts at the time of FN and baseline lymphocytes counts (Pearson's *r* = .38), suggesting that the lymphocyte counts to some extent reflect the prognosis of the underlying cancer. Both high CRP and low hemoglobin were prognostic the first year after FN, supporting a previous study where high CRP was associated with increased risk of short‐term medical complications after FN.^27^ Other studies have shown that the CRP levels at initiation of chemotherapy is a prognostic marker for overall survival in patients with cancer.^26^, ^28^ However, we found only a weak correlation between CRP levels at the time of FN and baseline CRP levels (Pearson's *r* = .21), possibly indicating that the CRP level at the time of FN reflects the severity of the FN event more than the severity of the underlying cancer. Low hemoglobin levels were also associated with an increased risk of mortality the first year after FN, a finding also found by Lyman et al (adjusted HR for overall mortality 1.43, 95% CI, 1.24‐1.64).^11^ This may reflect advanced disease stage due to the extent of chronic inflammation.^29^ Supporting this notion, we found a moderate correlation between anemia at the time of FN and baseline anemia (Pearson's *r* = .54). Moreover, cancer‐related anemia may increase susceptibility to infection, induce functional deficits in lymphocytes, and reduce antineoplastic efficacy,^29^ which could also explain the identified association. The risk factors identified in this study, especially CRP and hemoglobin levels, could with renewed interest be considered for inclusion in the MASCC and CISNE scores, since we found they were prognostic for longer than short term.

We found that the 38% increased risk of all‐cause mortality after FN was at least partially explained by a twofold increased risk of infectious mortality. Further, FN was associated with a twofold higher risk of ICU admissions, presumably associated with organ failures related to severe infection. Accordingly, our results indicate that prevention of FN could lead to lower mortality and morbidity, and we thus corroborate a meta‐analysis presenting a relative risk of all‐cause mortality of 0.93 (95% CI, 0.90‐0.96) in patients treated with prophylactic G‐CSF.^30^

We found a nonsignificant trend for increased risk of cardiovascular mortality after FN, congruous with a recent review on how acute infections are associated with increased cardiovascular mortality.^13^

The major strength of this study was the combination of nationwide clinical data generated through routine clinical care and data from the Danish health registries that facilitates almost complete ascertainment of outcomes and follow‐up.

A pivotal consideration of this study is that it is feasible to pool cancer types with varying baseline mortality rates and then assess the impact of FN on mortality in the pooled population. The ensuing heterogeneous population may diminish the confidence in interpretation of the associations between risk factors and mortality. However, we did not find that specific cancer types clustered according to the distribution of risk factors (results not shown). Another consideration was the broad categorization of patients according to the number of risk factors present at the time of FN, resulting in heterogeneous groups of patients. However, the wide differences in absolute mortality between the groups depicted in Figure 2 affirms our approach.

The main limitation of the study was the use of a nonconventional FN definition that did not include temperature measurements. However, we have previously shown good concordance between this definition and a guidelines‐based definition of FN of fever ≥38 degrees Celsius and neutropenia <0.5 × 10^9^/L.^15^, ^16^ Another limitation was that we included patients from only a single center and thus results may not be generalizable to other settings. However, patients at our institution are treated according to ESMO guidelines; hence, we do not believe this to influence results substantially. We were not able to compare our results with the MASCC and CISNE scores, due to the lack of data on risk factors included in these scores. Accordingly, we were not able to fully adjust for known risk factors, such as treatment intent, performance status, or involvement of the bone marrow, which we did not have data on, and albumin, glucose, and monocytes levels, which we had too much missingness for.

In summary, we found increased all‐cause and infectious mortality and risk of ICU admissions after FN in a large cohort of consecutive patients with several types of cancer. We further identified subgroups of patients with FN with a markedly increased risk of death associated with the presence of risk factors at the time of FN. Since the incidence of FN can be reduced by G‐CSF^31^ and, in some cases, prophylactic antibiotics,^32^ these results are important for clinicians, researchers, and policy makers.

## 5 DATA AVAILABILITY STATEMENT

The data that support the findings of this study are available from the corresponding author upon reasonable request

## CONFLICT OF INTEREST

There are no conflicts of interest related to this study. Professor Specht is a member of the advisory board and principal investigator for Takeda, a member of the advisory board for Merck, has a research agreement with Varian Medical Systems and Merck Serono, and is a principal investigator for Nanovi outside the submitted work. Professor Mocroft has received personal honoraria, travel support or consultancy fees from ViiV Healthcare and Gilead Sciences outside the submitted work. All remaining authors have declared no conflicts of interest.

## Supporting information

Data S1
