# Supplementary material for: Mortality and admission to intensive care units after febrile neutropenia in patients with cancer
Source: Cancer Med. 2020 Mar 7;9(9):3033–42. doi: 10.1002/cam4.2955 (PMC7196064; doi:10.1002/cam4.2955)
Supplement: Supplementary file 1 — Data S1 [file CAM4-9-3033-s001.doc]

Supplementary material for the manuscript: “Mortality and admission to intensive care units after febrile neutropenia in patients with cancer”

Authors: Aagaard T, Reekie J, Jørgensen M, Roen A, Daugaard G, Specht L, Sengeløv H, Mocroft A, Lundgren J, Helleberg M

Contents

[Assessment of cycle length 2](#__RefHeading___Toc28607846)

[Definitions of risk factors 2](#__RefHeading___Toc28607847)

[Infectious and cardiovascular mortality 4](#__RefHeading___Toc28607848)

[ICU admissions 5](#__RefHeading___Toc28607849)

[References 6](#__RefHeading___Toc28607850)

[Supplementary Tables 8](#__RefHeading___Toc28607851)

# **Assessment of cycle length**

Cycle length was used in assessment of chemotherapy dose delays and dose reductions and G-CSF prophylaxis.

Cycle length was based on the standard regimen schedules used in the Department of Oncology combined with information on when chemotherapy was received. The start date of the first cycle was defined as the first date of receiving chemotherapy in the chemotherapy course; we only used this date to assess the start date of the subsequent cycle as the first cycle was not included in the study. For each cycle, the start date was defined as the next date of receiving chemotherapy after the start date of the previous cycle, at the earliest one day shorter than the date the regimen schedule would designate. We defined the last day of the cycle as the day before first date of chemotherapy in the next cycle or, if no new cycle commenced, after standard cycle length for that regimen had passed.

# **Definitions of risk factors**

Data sources are meticulously described in the supplementary material to the FENCE study^1^.

**Charlson Comorbidity Index** was calculated on Day zero of the first cycle, using the coding algorithm presented by Quan et al.^2^ for the updated index by Quan et al.^3^ from all A- (primary) and B- (secondary) diagnoses from both inpatient and outpatient visits as registered in the LPR^4^ on or before the start date of the first cycle. Points attributed to the cancer was not counted. The Charlson Comorbidity Index has been validated in data from the LPR where the positive predictive value of the diagnoses found in the LPR was found to be 98% with a range of 88-100%^5^.

**Disease stage**: Chemotherapy regimens were considered 1) adjuvant, 2) neoadjuvant or concomitant or 3) locally advanced or disseminated based on the name of the chemotherapy regimen in PPAS as assessed by Theis Aagaard and validated by Gedske Daugaard.

**Body surface area** was calculated as close before the start of each cycle as possible, by the DuBois and DuBois method, BSA = 0.007184 * Height^0.725^ * Weight^0.425^ with height and weight extracted from PPAS and KISO^1^.

**Baseline anaemia** was identified as the sample closest to and up to 90 days before the start of chemotherapy at the latest on the start date. The following reference range for haemoglobin was used as reported from the Department of Biochemistry at Rigshospitalet, University of Copenhagen^6^: 11.8-15.3 g/dL for women and 13.4-16.9 g/dL for men

**Radiotherapy** was assessed by identifying the initiation and end dates of all courses of radiation therapy in ARIA9/ARIA11^1^, assessing if radiotherapy had been initiated any time prior to chemotherapy.

**Chemotherapy dose delay** was defined based on the standard cycle length for a regimen as extracted from the standard regimen schedules used at the Department of Oncology at Rigshospitalet, University of Copenhagen and counted in days. We assessed the risk factor as a binary time-updated variable, with a ≥15% delay^7^ compared to the previous cycle. The first dose delay was used as a marker of deviation from on-time chemotherapy.

**Chemotherapy dose reduction** was based on doses reported in PPAS and calculated as current dose/previous dose*100 at the initiation of each cycle, using the largest reduction for any of the drugs given. Chemotherapy given within cycles was not considered. If a new drug was commenced, the dose % compared to the standard for that regimen was extracted from PPAS if available, otherwise assumed to be one hundred %. We assessed the risk factor as a binary time-updated variable, with a ≥15% reduction^7^ compared to the previous cycle. The first dose delay was used as a marker of deviation from full-dose chemotherapy.

**Prophylactic G-CSF** was defined as G-CSF given on Day 1-7 of each cycle. Types of G-CSF included filgrastim, pegfilgrastim and lipegfilgrastim, with the clear majority being the latter two types. We assessed the first prescription of G-CSF as a marker of initiation and continued use of G-CSF.

# **Infectious and cardiovascular mortality**

Data on cause of death were available for the period 2010-2015. The categories of cause of death were assessed by the following ICD-10 codes in the Danish Register of Causes of Death^8^:

**Infectious**: A00-B899, B95-B999, D733, G00-G029, G04-G052, G06-G089, H100, H67-H671, H700, I30-I309, I320-I321, I33-I339, I38-I389, I400, I410-I412, I520-I521, I681, I980-I981, J00-J229, J32, J340, J350, J36-J379, J383, J387, J390-J391, J440, J85-J869, K35-K359, K570, K572, K574, K578, K61-K619, K630, K650, K67, K750, K770, K810, K930, K931, L00, L010, L02-L039, L08, M00-M019, M462-M463, M490-M493, M60, M726, N10-N109, N11-N129, N136, N151, N160, N300, N308, N309, N330, N34, N390, N41-N419, N45-N459, N70-N749, N751, N76-N771

**Cardiovascular:** I01-I99

# **ICU admissions**

ICU admissions were identified in the National Patient Register^4^. We used the following SKS department codes to identify intensive care units: 1301011, 1301012, 1301342, 130132C, 1303010, 1307010, 1309010, 1309610, 1330250, 1351100, 1401010, 1501012, 1502010, 1516011, 1516016, 2000341, 2501011, 2502010, 3800Q20, 5501010, 6630332, 7003011, and 7601010.

# **References**

1. Aagaard T, Roen A, Reekie J, Daugaard G, Brown P de N, Specht L, Sengeløv H, Mocroft A, Lundgren J, Helleberg M. Development and Validation of a Risk Score for Febrile Neutropenia After Chemotherapy in Patients With Cancer: The FENCE Score. *JNCI Cancer Spectr* 2018;2.

2. Quan H, Sundararajan V, Halfon P, Fong A, Burnand B, Luthi J-C, Saunders LD, Beck C a, Feasby TE, Ghali W a. Coding algorithms for defining comorbidities in ICD-9-CM and ICD-10 administrative data. *Med Care* 2005;43:1130–9.

3. Quan H, Li B, Couris CM, Fushimi K, Graham P, Hider P, Januel J-M, Sundararajan V. Updating and validating the Charlson comorbidity index and score for risk adjustment in hospital discharge abstracts using data from 6 countries. *Am J Epidemiol* 2011;173:676–82.

4. Lynge E, Sandegaard JL, Rebolj M. The Danish National Patient Register. *Scand J Public Health* 2011;39:30–3.

5. Thygesen SK, Christiansen CF, Christensen S, Lash TL, Sørensen HT. The predictive value of ICD-10 diagnostic coding used to assess Charlson comorbidity index conditions in the population-based Danish National Registry of Patients. *BMC Med Res Methodol* 2011;11:83.

6. Biochemistry reference ranges at Rigshospitalet, University of Copenhagen [Internet]. Available from: https://www.rigshospitalet.dk/afdelinger-og-klinikker/diagnostisk/klinisk-biokemisk-afdeling/for-fagfolk/Sider/analyseoplysninger-blegdamsvej.aspx

7. Crawford J, Dale DC, Kuderer NM, Culakova E, Poniewierski MS, Wolff D, Lyman GH. Risk and timing of neutropenic events in adult cancer patients receiving chemotherapy: the results of a prospective nationwide study of oncology practice. *J Natl Compr Canc Netw* 2008;6:109–18.

8. Helweg-Larsen K. The Danish Register of Causes of Death. *Scand J Public Health* 2011;39:26–9.

# **Supplementary Tables**

**Supplementary Table 1. Frequency of febrile neutropenia by chemotherapy regimen**

| Cancer type | Chemotherapy regimen | Patients treated, n | Patients with FN, n (%) |
| --- | --- | --- | --- |
| Ovarian | Docetaxel 75mg/m2 + carboplatin AUC5 q3w for six cycles | 486 | 124 (25.5) |
| Breast | Epirubicin 90mg/m2 + cyclophosphamide 600mg/m2 q3w for three cycles followed by docetaxel 100mg/m2 q3w for three cycles OR paclitaxel 80mg/m2 q1w for nine cycles +- tamoxifen/letrozole | 1063 | 114 (10.7) |
| Non-small-cell lung | Carboplatin AUC5 + oral vinorelbine 60mg/m2 day 1 AND oral vinorelbine 80mg/m2 day 8 q3w for four cycles | 430 | 74 (17.2) |
| Gastric | Docetaxel 60mg/m2 + Carboplatin AUC5 + 5FU 3200mg/m2 (continuous for 96h) q3w for six cycles | 365 | 77 (21.1) |
| Small-cell lung | Carboplatin AUC5 + oral etoposide 200mg/m2 day 1, 2, and 3 q3w for six cycles | 226 | 59 (26.1) |
| Testicular | Cisplatin 20mg/m2 day 1, 2, 3, 4, and 5 + etoposide 100mg/m2 day 1, 2, 3, 4, and 5 + bleomycin 15,000IU/m2 day 2, 8, and 15 q3w for three or four cycles | 316 | 55 (17.4) |
| Prostate | Docetaxel 75mg/m2 q3w for six to ten cycles | 248 | 35 (14.1) |
| Neuroendocrine | Carboplatin AUC5 + oral etoposide 200mg/m2 day 1, 2, and 3 q3w for six cycles | 249 | 42 (16.9) |

Only regimens with febrile neutropenia n>30 are shown
